# Randomized trial promoting cancer genetic risk assessment when genetic counseling cost removed: 1-year follow-up

**DOI:** 10.1093/jncics/pkae018

**Published:** 2024-03-15

**Authors:** Jinghua An, Jean McDougall, Yong Lin, Shou-En Lu, Scott T Walters, Emily Heidt, Antoinette Stroup, Lisa Paddock, Sherry Grumet, Deborah Toppmeyer, Anita Y Kinney

**Affiliations:** Rutgers Cancer Institute of New Jersey, New Brunswick, NJ, USA; Fred Hutchinson Cancer Center, Seattle, WA, USA; Rutgers Cancer Institute of New Jersey, New Brunswick, NJ, USA; Rutgers University School of Public Health, Piscataway, NJ, USA; Rutgers Cancer Institute of New Jersey, New Brunswick, NJ, USA; Rutgers University School of Public Health, Piscataway, NJ, USA; University of North Texas Health Science Center, Fort Worth, TX, USA; Rutgers Cancer Institute of New Jersey, New Brunswick, NJ, USA; Rutgers Cancer Institute of New Jersey, New Brunswick, NJ, USA; Rutgers University School of Public Health, Piscataway, NJ, USA; Rutgers Cancer Institute of New Jersey, New Brunswick, NJ, USA; Rutgers University School of Public Health, Piscataway, NJ, USA; Rutgers Cancer Institute of New Jersey, New Brunswick, NJ, USA; Rutgers Cancer Institute of New Jersey, New Brunswick, NJ, USA; Rutgers Cancer Institute of New Jersey, New Brunswick, NJ, USA; Rutgers University School of Public Health, Piscataway, NJ, USA

## Abstract

**Purpose:**

Cancer genetic risk assessment (CGRA) is recommended for women with ovarian and high-risk breast cancer. However, the underutilization of CGRA has long been documented, and cost has been a major barrier. In this randomized controlled trial, a tailored counseling and navigation (TCN) intervention significantly improved CGRA uptake at 6-month follow-up, compared with targeted print (TP) and usual care (UC). We aimed to examine the effect of removing genetic counseling costs on CGRA uptake by 12 months.

**Methods:**

We recruited racially and geographically diverse women with breast and ovarian cancer from cancer registries in Colorado, New Jersey, and New Mexico. Participants assigned to TCN received telephone-based psychoeducation and navigation. After 6 months, the trial provided free genetic counseling to participants in all arms.

**Results:**

At 12 months, more women in TCN obtained CGRA (26.6%) than those in TP (11.0%; odds ratio [OR] = 2.77, 95% confidence interval [CI] = 1.56 to 4.89) and UC (12.2%; OR = 2.46, 95% CI = 1.41 to 4.29). There were no significant differences in CGRA uptake between TP and UC. The Kaplan-Meier curve shows that the divergence of cumulative incidence slopes (TCN vs UC, TCN vs TP) appears primarily within the initial 6 months.

**Conclusion:**

TCN significantly increased CGRA uptake at the 12-month follow-up. Directly removing the costs of genetic counseling attenuated the effects of TCN, highlighting the critical enabling role played by cost coverage. Future policies and interventions should address multilevel cost-related barriers to expand patients’ access to CGRA.

**Trial Registration:**

This trial was registered with the NIH clinical trial registry, clinicaltrials.gov, NCT03326713. https://clinicaltrials.gov/ct2/show/NCT03326713.

Germline pathogenic variants in genes, such as *BRCA1*, *BRCA2*, and *PALB2*, account for most hereditary breast and ovarian cancers (HBOC) (1,2). Identifying HBOC is crucial for the prevention and early detection of a second cancer and guiding treatment options in cancer patients (3-8). It can also trigger cancer prevention and risk management actions in their at-risk biological relatives (1). The most efficient way to identify women with HBOC pathogenic variants is to test those with a diagnosis of ovarian, fallopian tube, peritoneal, or high-risk breast cancers (9,10). Cancer genetic risk assessment (CGRA) is recommended for these women according to national guidelines (9,10). CGRA is a consultation service that includes clinical assessment of hereditary cancer risk, genetic testing when appropriate, and risk management recommendations in one or more genetic counseling sessions (11). CGRA is vital for helping eligible cancer survivors understand their risks for hereditary cancer and adopt recommended cancer risk management strategies (12,13).

Although the clinical utility of CGRA (ie, genetic counseling and/or genetic testing) is well established, more than half of breast and ovarian cancer survivors who meet national criteria have not had a CGRA (14-16). This underutilization is especially common among rural residents and racial and ethnic minorities (17-22). Facilitating CGRA uptake is complex, as it requires addressing structural (eg, cost, lack of physician recommendation or referral) and individual barriers (eg, lack of awareness of CGRA benefits) (22-25).

Although the cost of clinical genetic services has decreased over recent years, many people continue to experience financial barriers (25-27). Under the Patient Protection and Affordable Care Act, most private insurers are required to pay for HBOC genetic counseling and *BRCA1/2* testing in cancer patients who are at high risk (9,28,29). However, the actual coverage for genetic counseling and testing varies by insurance type, medical facility, and testing laboratory (29-31). In the United States, additional barriers include low awareness of HBOC risks and CGRA (17,32-34), anticipated negative emotional reactions (32,33,35), and logistical difficulties (33,34,36).

In the Genetic Risk Assessment for Cancer Education and Empowerment Project, a telephone-based tailored counseling and navigation (TCN) intervention was designed to increase CGRA in ethnically and geographically diverse cancer survivors who met the National Comprehensive Cancer Network’s (NCCN’s) criteria for identifying HBOC (10). A “traceback” strategy was adopted to identify women who had not received testing at the time of their diagnosis and were thus unaware of their elevated risk. The tailored intervention incorporated health communication and behavior change theories alongside motivational interviewing strategies to engage women in seeking CGRA. Large effects were observed after 6 months following the intervention: more women in TCN received CGRA (18.7%) than those in the nontailored targeted print (TP) arm (3%; odds ratio [OR] = 7.4, 95% confidence interval [CI] = 3.0 to 18.3) or the usual care (UC) arm (2.5%; OR = 8.9, 95% CI = 3.4 to 23.5) (26). Nevertheless, the fact that only 1 in 5 participants sought CGRA at 6 months in the TCN arm suggested the existence of persistent barriers, including cost concerns (real or perceived) and lack of referral by a clinician (26). To help remove cost barriers after the 6-month follow-up, we informed all participants who reported not having had genetic counseling or testing that the study would provide genetic counseling at no cost to them. Although the results of the primary outcome analysis were previously reported (26,37,38), this article focuses on CGRA uptake at the 12-month follow-up (ie, the key secondary outcome). In this study, we aimed to test the effect of TCN on the behavioral outcome (CGRA uptake) at the 12-month follow-up when genetic counseling fees were covered by the study.

## Methods

### Study design

A 3-arm randomized superiority trial was conducted in women with ovarian or high-risk breast cancer. The trial design, development, and implementation were described elsewhere (26,39) and approved by the participating institutions’ Institutional Review Boards. After informed consent and baseline survey completion, participants were randomized into 1 of the 3 arms (1:1:1) using a computer-generated random number list with a size of 9 in each block. We report this trial following the recommended standards of the extended Consolidated Standards of Reporting Trials statement for parallel-group, nonpharmacologic randomized trials (40), and the trial was registered on clinicaltrials.gov #NCT03326713 (41).

### Participants and sample size

Women were recruited from 3 statewide cancer registries in New Jersey, Colorado, and New Mexico between November 2017 and July 2020. To be eligible, participants had to be biologically female, age 21 years or older, be fluent in English or Spanish, and meet at least one of the NCCN criteria for CGRA (breast cancer diagnosed at age 50 years or younger; triple-negative breast cancer diagnosed at age 60 years or older; 2 or more primary breast cancers; and/or any epithelial ovarian, peritoneal, or fallopian tube cancer). Women were excluded if they had previous genetic counseling or testing for HBOC, had no access to a telephone, or planned to move out of 1 of the 3 states within 1 year. *A priori* power analysis was conducted for the primary outcome, 6-month CGRA uptake (26). In comparing the CGRA uptake at 12 months in this article, and given the sample size of at least 212 participants per arm, the *post hoc* power analysis showed that the minimum detectable odds ratio for comparisons between TCN and TP or between TCN and UC was 2.3 with 80% power and 5% overall type I error rate (2.5% for each comparison after multiplicity adjustment), on the basis of the 12-month CGRA rate of 11%-12% for TP or UC.

### Study arms and interventions

Participants in the TP arm received an educational brochure within 1 week of completing the baseline survey. The brochure content was guided by health communication and behavioral change theories (42-44). Presented in English and Spanish, the brochure addressed knowledge about HBOC risks, threat appraisal (to validate or raise perceived seriousness and risks of HBOC), response efficacy (benefits and expectations about CGRA), and self-efficacy (CGRA facilities, cost, and insurance reimbursement).

No intervention was provided to the UC arm in the initial 6 months. After completing the 6-month assessment, participants in this arm received the educational brochure.

Women in the TCN arm were mailed the same brochure and a sealed envelope containing visual aids upon completing the baseline survey. Subsequently, they were engaged in a 30- to 45-minute tailored telephone call with a health coach within 2 weeks. Guided by health communication and behavioral change theories, the coaches raised participants’ perceived threat of HBOC and efficacy in averting the threat, as well as assisting in formulating an action plan. The session was tailored according to individual characteristics (eg, perceived HBOC risk, response efficacy of CGRA, self-efficacy, barriers and concerns, nearby cancer genetic providers, and personal and family cancer history). All coaches were trained and supervised in motivational interviewing. Participants were reminded of their action plan in a follow-up tailored letter (sent immediately after the telephone call) and in a mailed Action Plan Reminder Card (6 weeks after the call). They also received another phone call after approximately 7 weeks to receive additional navigation if needed. In addition, when participants gave permission, a letter was sent to their providers to inform them about their patient’s eligibility for a genetics referral.

After the 6-month survey, a letter informing patients that the study would cover the cost of genetic counseling was sent to participants in all study arms who reported that they had not completed genetic counseling or testing (hereinafter, the cost-coverage letter). Participants were asked to contact the study staff within 3 weeks to be connected to free genetic counseling services and to obtain the services within 3 months upon receiving the cost-coverage letter.

### Data collection and measures

Telephone or online surveys (per patient preference) were administered at baseline, 1 month, 6 months, and 12 months after the interventions for the TP and TCN arms and the baseline survey for the UC arm. Cancer diagnosis was obtained from the cancer registries; other eligibility criteria were collected by self-report. Demographic and clinical variables (eg, family cancer history) were assessed at baseline in surveys. Participants reported whether they had sought cancer CGRA (ie, genetic counseling and/or testing) in surveys at the 6-month and 12-month follow-ups. Participants who reported receiving a CGRA were asked to provide written consent allowing research staff to obtain documentation of receipt of the genetic services. Health literacy (45) was assessed with a validated measure that is used to screen for people with inadequate health literacy (α = .74). Participants were followed for 12 months or until death or withdrawal of consent. Data collectors were blinded to study arm assignment.

### Statistical analysis

An intent-to-treat principle was followed in the analysis. The demographic and clinical characteristics of participants were compared across the 3 arms using analysis of variance and χ^2^ analysis. The analysis of CGRA uptake at 6 months has been reported elsewhere (26). Logistic regression analysis was employed to compare the medically verified CGRA uptake (yes/no) at 12 months between TCN vs UC and TP; a binary intervention variable (TCN vs TP; TCN vs UC) was added to logistic models separately. Negative outcome imputation and multiple imputation were then used as sensitivity analyses to determine whether the intervention effect estimates were sensitive to imputation and if the conclusions were consistent. Negative outcome imputation assumed that CGRA did not occur if there was no documented verification. Multiple imputation was based on verified CGRA within 12 months and demographic and clinical variables, including age, race and ethnicity, marital status, health insurance status, education level, health literacy level, employment status, household income, rural/urban residence, having a primary care provider, cancer site (breast vs ovarian cancer), and number of first- and second-degree relatives with cancer. Twenty-five imputed datasets were used to provide a combined estimate for missing values based on Rubin’s rule (46), using Proc MI and Proc MIANLYZE in SAS v9.4. To estimate the cumulative probability of medically verified CGRA uptake following the intervention to 12-month follow-up, Kaplan-Meier estimates were calculated along with the 95% confidence intervals for each study arm. The event-of-interest was receiving genetic counseling or testing, whichever was earlier. Participants were censored if they were lost to follow-up or the event did not occur within the 12-month follow-up. All tests of statistical significance were 2-sided.

## Results

**Figure 1.**
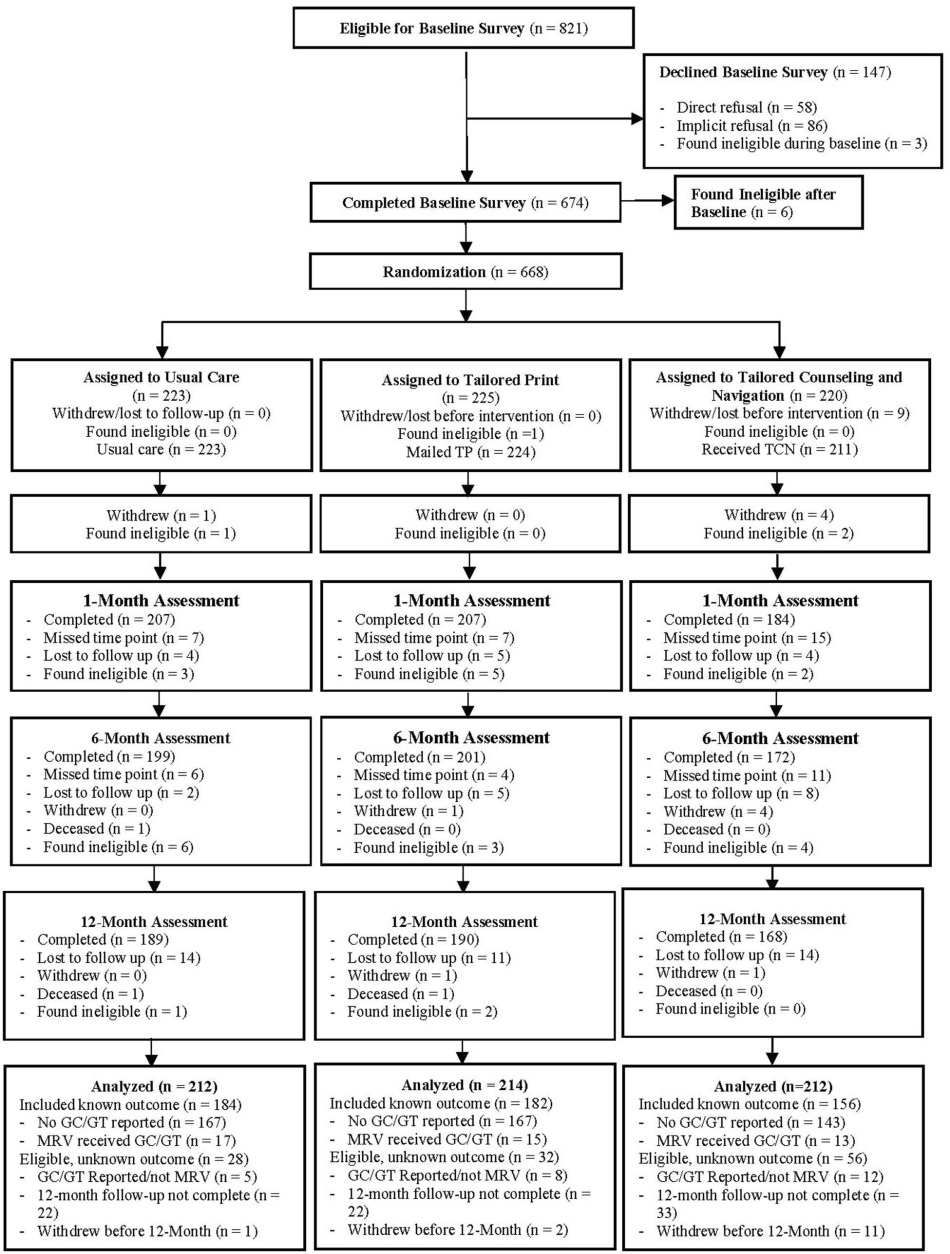
GRACE study CONSORT diagram. In the intent-to-treat analysis, only participants found ineligible after random assignment were excluded. GRACE = Genetic Risk Assessment for Cancer Education and Empowerment; GC/GT = genetic counseling and/or testing; MRV = medical record verified; TCN = tailored counseling and navigation; TP = targeted print.

Of those deemed eligible at the initial assessment (n = 821), a total of 668 participants were randomly assigned to 3 arms; 30 were found to be ineligible after random assignment because they were later found to have had a CGRA before the study. Thus, 638 women were included in the analysis. The study enrollment, randomized assignment, and retention summary are presented in Figure 1. The 12-month retention rate was 87.9%, with no statistically significant differences between participants in each arm (*P* = .99). Participants in the 3 arms did not differ in baseline demographic or clinical characteristics except for language preferences (Table 1). High intervention fidelity in the TCN arm was evidenced by 95% of coaching sessions compliant with the fidelity checklist; key indicators of motivational interviewing fidelity (eg, global scores, % complex reflections) were consistently in the good-to-excellent range.

**Table 1.** Sociodemographic and clinical characteristics of participants by study arm

Study arm	All, n (%) (N = 638)	UC, n (%) (n = 212)	TP, n (%) (n = 214)	TCN, n (%) (n = 212)	*P*
Age (mean, SD)	61.1 (10.2)	61.1 (10.0)	61.0 (10.1)	61.2 (10.7)	.99
Years since diagnosis (mean, SD)	11.2 (7.7)	11.3 (7.7)	10.9 (7.6)	11.3 (7.7)	.84
Self-reported race and ethnicity					
Hispanic	159 (25.4)	55 (26.6)	46 (21.8)	58 (27.9)	.40
Non-Hispanic White	377 (60.2)	128 (61.8)	130 (61.6)	119 (57.2)	
Non-Hispanic Black	37 (5.9)	7 (3.4)	17 (8.1)	13 (6.3)	
Other^a^	53 (8.5)	17 (8.2)	18 (8.5)	18 (8.7)	
Missing	12	5	3	4	
Self-reported Ashkenazi Jewish ancestry					
No	594 (97.1)	197 (97.0)	201 (96.2)	196 (98.0)	.55
Yes	18 (2.9)	6 (3.0)	8 (3.8)	4 (2.0)	
Missing	26	9	5	12	
Marital status					
Single/Divorced/Separated/Widowed	249 (39.1)	76 (35.8)	88 (41.3)	85 (40.1)	.48
Married/Domestic partnership	388 (60.9)	136 (64.2)	125 (58.7)	127 (59.9)	
Missing	1	0	1	0	
Education level					
Less than high school/High school grad/GED	115 (18.2)	43 (20.4)	40 (18.9)	32 (15.3)	.33
Some college, assoc. degree, or vocational school	227 (35.9)	80 (37.9)	67 (31.6)	80 (38.3)	
Bachelor’s degree or higher	290 (45.9)	88 (41.7)	105 (49.5)	97 (46.4)	
Missing	6	1	2	3	
Annual household income					
<$30,000	140 (24.7)	51 (27.1)	43 (22.6)	46 (24.5)	.92
$30,000-$49,999	101 (17.8)	35 (18.6)	35 (18.4)	31 (16.5)	
$50,000-$69,999	85 (15.0)	29 (15.4)	28 (14.7)	28 (14.9)	
$70,000 or more	240 (42.4)	73 (38.8)	84 (44.2)	83 (44.1)	
Missing	72	24	24	24	
Health literacy level					
Adequate (<9)	624 (98.0)	208 (97.7)	208 (98.1)	208 (98.1)	.93
Marginal/Inadequate (≥9)	13 (2.0)	5 (2.3)	4 (1.9)	4 (1.9)	
Missing	1	1	0	0	
Rural/urban residence^b^					
Urban	526 (82.4)	184 (86.8)	173 (80.8)	169 (79.7)	.12
Rural	112 (17.6)	28 (13.2)	41 (19.2)	43 (20.3)	
Has health insurance					
No	34 (5.3)	15 (7.1)	7 (3.3)	12 (5.7)	.21
Yes	602 (94.7)	197 (92.9)	207 (96.7)	198 (94.3)	
Missing	2	0	0	2	
Has a personal health-care provider					
No	26 (4.1)	9 (4.2)	8 (3.7)	9 (4.2)	.95
Yes	612 (95.9)	203 (95.8)	206 (96.3)	203 (95.8)	
Cancer site					
Ovarian	94 (14.7)	36 (17.0)	32 (15.0)	26 (12.3)	.39
Breast	544 (85.3)	176 (83.0)	182 (85.0)	186 (87.7)	
Number of FDR and SDR with breast or ovarian cancer					
0 FDR and 0 SDR	406 (63.6)	131 (61.8)	140 (65.4)	135 (63.7)	.84
1 FDR or 1 SDR	131 (20.5)	49 (23.1)	40 (18.7)	42 (19.8)	
2 or more FDR/SDR	101 (15.8)	32 (15.1)	34 (15.9)	35 (16.5)	
Years since diagnosis					
<5	164 (25.8)	51 (24.2)	59 (27.7)	54 (25.5)	.75
≥5 to <10	151 (23.7)	56 (26.5)	45 (21.1)	50 (23.6)	
≥10	321 (50.5)	104 (49.3)	109 (51.2)	108 (50.9)	
Missing	2	1	1	0	
Informed of increased HBOC risk by a healthcare provider					.54
No	385 (60.4)	133 (62.7)	130 (61.0)	122 (57.6)	
Yes	252 (39.6)	79 (37.3)	83 (39.0)	90 (42.5)	
Missing	1	0	1	0	
Language preference					
English	611 (95.8)	196 (92.5)	211 (98.6)	204 (96.2)	.006
Spanish	27 (4.2)	16 (7.5)	3 (1.4)	8 (3.8)	

a “Other” refers to American Indian or Alaska Native, Asian American, and Pacific Islander. FDR = first-degree relatives; HBOC = hereditary breast and ovarian cancer; SDR = second-degree relatives; SD = standard deviation; TCN = tailored counseling and navigation; TP = targeted print; UC = usual care.

b Rural or urban residence was based on Rural-Urban Commuting Area codes at the ZIP code level.

Of the 89 women who had CGRA, 12 (13.5%) had pretest counseling only, 26 (29.2%) had genetic testing only, and 51 (57.3%) had both genetic counseling and testing by the 12-month follow-up (Table 2). A higher percentage of CGRA in the TCN arm was consistently observed, regardless of imputation methods (Table 3). Logistic regression (Table 4) showed that the rate of medical record-verified CGRA uptake was higher in participants randomly assigned to the TCN arm than in the UC arm (OR = 2.46, 95% CI = 1.41 to 4.29) and TP arm (OR = 2.77, 95% CI = 1.56 to 4.89). CGRA rates were not statistically different between TP and UC arms. The pattern was consistently observed in different imputation scenarios (Table 4).

**Table 2.** Characteristics of medically verified CGRA within 12 months^a^

Study arm	All, n (%) (N = 638)	UC, n (%) (n = 212)	TP, n (%) (n = 214)	TCN, n (%) (n = 212)
CGRA by months				
Had CGRA in 0-6 months	43 (7.8)	5 (2.6)	6 (3.1)	32 (18.9)
Had CGRA 6-12 months	46 (8.4)	18 (9.5)	15 (7.9)	13 (7.7)
Had no CGRA	460 (83.8)	166 (87.8)	170 (89.0)	124 (73.4)
Missing	89	23	23	43
Type of services obtained				
Pretest counseling only	12 (2.2)	1 (0.5)	2 (1.0)	9 (5.3)
Genetic testing only	26 (4.7)	7 (3.7)	3 (1.6)	16 (9.5)
Both genetic counseling and testing	51 (9.3)	15 (7.9)	16 (8.4)	20 (11.8)
No genetic counseling or testing	460 (83.8)	166 (87.8)	170 (89.0)	124 (73.4)
Missing	89	23	23	43

a CGRA = cancer genetic risk assessment; TCN = tailored counseling and navigation; TP = targeted print; UC = usual care.

**Table 3.** Self-reported and medically verified CGRA uptake by study arms^a^

Study arm	All, n (%) (N = 638)	UC, n (%) (n = 212)	TP, n (%) (n = 214)	TCN, n (%) (n = 212)
Self-reported CGRA				
Outcome known				
Had CGRA	76 (13.9)	19 (10.1)	21 (11.1)	36 (21.4)
No CGRA	470 (86.1)	169 (89.9)	169 (88.9)	132 (78.6)
Missing	92	24	24	44
% CGRA (95% CI)	13.9 (11.0 to 16.8)	10.1 (5.8 to 14.4)	11.1 (6.6 to 15.5)	21.4 (15.2 to 27.6)
Negative outcome imputation				
Had CGRA	76 (11.9)	19 (9.0)	21 (9.8)	36 (17.0)
No CGRA/Did not report	562 (88.1)	193 (91.0)	193 (90.2)	176 (83.0)
% CGRA (95% CI)	11.9 (9.4 to 14.4)	9.0 (5.1 to 12.8)	9.8 (5.8 to 13.8)	17.0 (11.9 to 22.0)
Multiple imputation				
Had CGRA	89.8 (14.1)	23.0 (10.8)	24.5 (11.4)	42.4 (20.0)
No CGRA/Did not report	548.2 (85.9)	189.0 (89.2)	189.5 (88.6)	169.6 (80.0)
% CGRA (95% CI)	14.1 (11.4 to 16.8)	10.8 (6.7 to 15.0)	11.4 (7.2 to 15.7)	20.0 (14.6 to 25.4)
Medical record verified CGRA				
Outcome known				
CGRA	89 (16.2)	23 (12.2)	21 (11.0)	45 (26.6)
No CGRA	460 (83.8)	166 (87.8)	170 (89.0)	124 (73.4)
Missing	89	23	23	43
% CGRA (95% CI)	16.2 (13.1 to 19.3)	12.2 (7.5 to 16.8)	11.0 (6.6 to 15.4)	26.6 (20.0 to 33.3)
Negative outcome imputation				
CGRA	89 (13.9)	23 (10.8)	21 (9.8)	45 (21.2)
No CGRA/Did not report	549 (86.1)	189 (89.2)	193 (90.2)	167 (78.8)
% CGRA (95% CI)	13.9 (11.3 to 16.6)	10.8 (6.7 to 15.0)	9.8 (5.8 to 13.8)	21.2 (15.7 to 26.7)
Multiple imputation				
CGRA	102.8 (16.1)	26.8 (12.6)	25.2 (11.8)	50.9 (24.0)
No CGRA/Did not report	535.2 (83.9)	185.2 (87.4)	188.8 (88.2)	161.1 (76.0)
% CGRA (95% CI)	16.1 (13.3 to 19.0)	12.6 (8.2 to 17.1)	11.8 (7.5 to 16.1)	24.0 (18.3 to 29.8)

a CGRA = cancer genetic risk assessment (genetic counseling and/or testing); CI = confidence interval; TCN = tailored counseling and navigation; TP = targeted print; UC = usual care.

**Table 4.** Logistic regression model for intervention effects on medically verified CGRA within 12 months

Model^a^	TCN vs TP	TCN vs UC	TP vs UC
Outcome known			
OR	2.77	2.46	0.89
95% CI	(1.56 to 4.89)	(1.41 to 4.29)	(0.47 to 1.67)
*P*	<.001	.002	.71
Negative outcome imputation^b^			
OR	2.35	2.07	0.88
95% CI	(1.34 to 4.12)	(1.20 to 3.59)	(0.47 to 1.65)
*P*	.003	.01	.69
Multiple imputation^c^			
OR	2.37	2.20	0.93
95% CI	(1.34 to 4.16)	(1.26 to 3.84)	(0.50 to 1.72)
*P*	.003	.006	.81

a Each of the 3 separate models (outcome known, negative outcome imputation, and multiple imputation) for pairwise comparisons of the 3 study arms. CGRA = cancer genetic risk assessment (genetic counseling and/or testing); CI =  confidence interval; TCN = tailored counseling and navigation; TP = targeted print; UC = usual care.

b Negative outcome imputation treated unknown CGRA outcome as no CGRA.

c Average number of CGRAs on the basis of 25 imputation datasets generated by IVEware52 with results combined by Proc MIANALYIZE using SAS 9.4.

**Figure 2.**
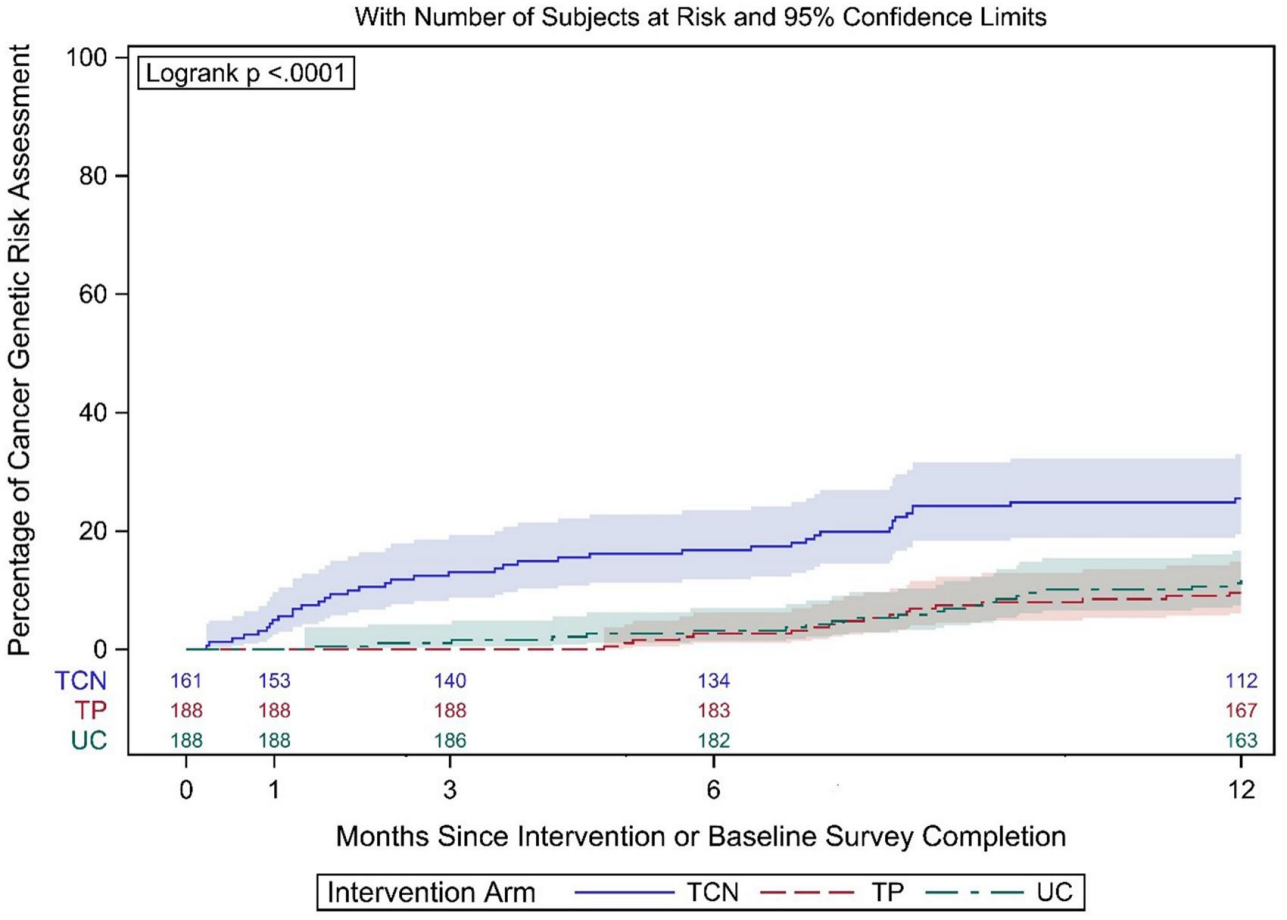
Cumulative incidence based on Kaplan-Meier estimates of CGRA uptake after intervention and 95% confidence intervals. CGRA = cancer genetic risk assessment (genetic counseling and/or testing); TCN = tailored counseling and navigation; TP = targeted print; UC = usual care.

Forty-three participants obtained CGRA in 0-6 months, and 46 obtained CGRA after receiving a cost-coverage letter (Table 2). As shown in the cumulative incidence curves (Figure 2), the TCN arm had a higher CGRA uptake throughout the 12-month follow-up period. The divergence of cumulative incidence slopes between the TCN vs UC and TP arms appears primarily within the first 6 months of the intervention. The difference between the slopes (TCN vs UC, TCN vs TP) appears to be sustained between 6 and 12 months.

## Discussion

As we and others have shown, the cost of genetic counseling remains an important barrier for cancer patients at elevated risks for HBOC (15,26,47,48) and is an important driver of disparities in cancer genomics care (25,49). To our knowledge, this is the first population-based trial to examine the impact of removing genetic counseling-related costs on CGRA uptake. After we sent the cost-coverage letter, the number of participants who sought CGRA doubled overall, suggesting that addressing cost barriers to genetic counseling is an important enabling factor.

At the 12-month follow-up, TCN significantly increased genetic counseling and/or testing uptake, compared with UC and TP (participants in UC and TP received the same intervention by 12 months). The TCN’s effectiveness and potential mechanisms have been reported elsewhere (26,37). Guided by behavioral and risk communication theories, the TCN intervention effectively enhanced participants’ knowledge and perceived susceptibility to HBOC, and self-efficacy to obtain CGRA, which led to strengthened CGRA intention and, ultimately, CGRA uptake (26,37). Compared with TCN’s effects on CGRA uptake at 6-month follow-up (26), the effect size at 12 months was attenuated by the cost-coverage letter: the odds ratio of having a CGRA decreased from 8.9 (95% CI = 3.4 to 23.5) to 2.46 for TCN vs UC, and from 7.4 (95% CI = 3.0 to 18.3) to 2.77 for TCN vs TP. The substantial decrease in odds ratios highlights the importance of eliminating cost barriers to genetic counseling.

Moreover, eliminating structural barriers, particularly cost barriers, is important in addition to improving patients’ awareness about hereditary cancers and genetic services (50). This was supported by the fact that only a very small proportion (3.1%) of TP participants sought CGRA within 6 months after receiving the educational brochure, whereas 7.9% of them sought CGRA after the cost-coverage letter was sent. Also, 18.9% and 7.7% of TCN participants had CGRA before and after receiving the cost-coverage letter, respectively, indicating the cost-coverage letter likely did not markedly amplify the TCN’s effects during the 6- to 12-month time frame. It might be because the navigation services of TCN helped overcome cost-related barriers during the initial 6 months, and/or that the importance of CGRA was effectively internalized for participants in the TCN arm (37).

Cost coverage by many third-party payers for CGRA has greatly improved in the past few decades, but a complex interaction between patient-, system-, and policy-level factors still produces significant cost barriers for both genetic counseling and testing for many eligible cancer patients (48,51-53). At the system and policy levels, coverage of genetic counseling and testing varies by insurers and medical institutions, and the coverage criteria do not always align with NCCN guidelines (30,52). For instance, Medicare requires genetic counseling with a genetic counselor to be billed only under physician supervision (30,53,54), forcing some patients to pay out of pocket. Medicaid covers genetic counseling and testing for HBOC in most states, but the specific requirements and limits vary by state (28). Moreover, genetic counseling is less likely to be reimbursed than genetic testing; the latter is more often covered by insurance and patient assistance programs (47,48). Some clinics and hospitals choose to not bill counseling (ie, providing free counseling to patients), but this may lead to underfinanced genetic counseling programs, worsening the shortage of genetic providers (55). Additionally, billing restrictions on tele-genetic counseling, including across state lines, are a commonly cited barrier to the implementation of telehealth (56,57). These system-level and policy-level hurdles need to be rectified by key stakeholders and policymakers.

At the patient level, patients are more willing to pay out-of-pocket costs (eg, cost share) for a genetic test than for genetic counseling (48). In addition, understanding insurance coverage details and “real” out-of-pocket costs can be a major concern even if the actual cost may be low for some insured patients (34,48,53). Even modest cost-sharing requirements can be burdensome for some patients (25).

Our intervention not only improved patients’ knowledge of cancer risk and perceived risk and efficacy beliefs (26,37), but also expanded their access to CGRA by providing cost coverage. However, we note that only 8% (46 out of 595) of participants took advantage of free genetic counseling by 12 months. Had women been provided free counseling when they were diagnosed, more might have taken advantage of it, but many of our participants were long-term cancer survivors and genetic testing was much more expensive at the time of their diagnosis (58). On the other hand, seeking CGRA can be complex as multiple steps and appointments are often required. Our centralized health coaches were not able to directly arrange CGRA for patients. Also, not directly providing cost coverage of genetic testing may also have contributed to the relatively low CGRA uptake rates. Our study provided free in-person or tele-genetic counseling services through 2 comprehensive cancer institutes or paid for genetic counseling if participants sought the service elsewhere. For participants who expressed cost concerns for testing, they were encouraged to use their participation compensation ($150) to help defray their out-of-pocket expenses and were connected to financial assistance programs. Overall, the relatively low CGRA uptake rate (16.2% across 3 arms) indicated that additional important barriers to CGRA still need to be addressed (eg, shortage of genetic counselors). Future research should more effectively examine the interplay between multilevel barriers. This low rate also suggested that the standard approach of referral to pretest genetic counseling may be a barrier to receiving guideline-concordant genetic testing. Thus, effective alternative care delivery models to pretest genetic counseling need to be implemented to fully enable survivors to obtain genetic testing (59).

Our study has some limitations. We were unable to assess the direct effect of cost coverage for genetic counseling alone because of the lack of a concurrent control group that was not offered free genetic counseling. However, our analysis approach elicited the impact of eliminating cost barriers for genetic counseling. Our study was also not powered to conduct a meaningful subgroup analysis to determine the impact of eliminating cost barriers on CGRA uptake among racial minorities, socioeconomic status, and insurance status. Additionally, we did not assess perceived costs; future research should address this issue. However, we examined participants’ CGRA-related out-of-pocket expenses and conducted a cost-effectiveness analysis, which will be reported elsewhere. Finally, most of our sample identified as non-Hispanic White and Hispanic/Latinx, limiting the generalizability of these findings to Black Americans and other racial minority patients.

Directly removing cost barriers to genetic counseling facilitates access to potentially lifesaving cancer genetic services. Future policies and interventions should more effectively target the challenges in accessing CGRA. This includes improving insurance reimbursement and lowering or removing out-of-pocket payments for both genetic counseling and testing.

## Data Availability

Deidentified data from this study are not available in a public archive under the current consent form.
